# The Klebsiella pneumoniae *ter* Operon Enhances Stress Tolerance

**DOI:** 10.1128/iai.00559-22

**Published:** 2023-01-18

**Authors:** Sophia Mason, Jay Vornhagen, Sara N. Smith, Laura A. Mike, Harry L. T. Mobley, Michael A. Bachman

**Affiliations:** a Department of Pathology, Michigan Medicine, University of Michigan, Ann Arbor, Michigan, USA; b Department of Microbiology & Immunology, Michigan Medicine, University of Michigan, Ann Arbor, Michigan, USA; c Department of Medical Microbiology & Immunology, University of Toledo, Toledo, Ohio, USA; University of California, Davis

**Keywords:** *Klebsiella*, tolerance, transposons, urinary tract infection

## Abstract

Healthcare-acquired infections are a leading cause of disease in patients that are hospitalized or in long-term-care facilities. Klebsiella pneumoniae (Kp) is a leading cause of bacteremia, pneumonia, and urinary tract infections in these settings. Previous studies have established that the *ter* operon, a genetic locus that confers tellurite oxide (K_2_TeO_3_) resistance, is associated with infection in colonized patients. Rather than enhancing fitness during infection, the *ter* operon increases Kp fitness during gut colonization; however, the biologically relevant function of this operon is unknown. First, using a murine model of urinary tract infection, we demonstrate a novel role for the *ter* operon protein TerC as a bladder fitness factor. To further characterize TerC, we explored a variety of functions, including resistance to metal-induced stress, resistance to radical oxygen species-induced stress, and growth on specific sugars, all of which were independent of TerC. Then, using well-defined experimental guidelines, we determined that TerC is necessary for tolerance to ofloxacin, polymyxin B, and cetylpyridinium chloride. We used an ordered transposon library constructed in a Kp strain lacking the *ter* operon to identify the genes that are required to resist K_2_TeO_3_-induced and polymyxin B-induced stress, which suggested that K_2_TeO_3_-induced stress is experienced at the bacterial cell envelope. Finally, we confirmed that K_2_TeO_3_ disrupts the Kp cell envelope, though these effects are independent of *ter*. Collectively, the results from these studies indicate a novel role for the *ter* operon as a stress tolerance factor, thereby explaining its role in enhancing fitness in the gut and bladder.

## INTRODUCTION

Klebsiella pneumoniae (Kp) is a pathogenic member of the *Enterobacteriaceae* family (order Enterobacterales) and a cause of pneumonia, UTI, and bloodstream infections (1). Importantly, Kp has a high potential for antibiotic resistance and is the third leading cause of global deaths that are attributable to pathogens with the potential for antibiotic resistance (2). Infection with antibiotic-resistant Kp is associated with high mortality rates, with the mortality rates ranging from approximately 20 to 40% (3–5). Multiple studies have demonstrated a strong association (odds ratio of approximately 4.0) between gut colonization and infection, indicating that infectious Kp originates from the guts of colonized patients (6–9). Colonization rates are variable, ranging as high as >75% in hospitalized patients (10), though several large studies indicate a colonization rate closer to 20% (6–8), depending on seasonal effects (11). Therefore, Kp can successfully and silently tolerate the hostile gut environment before causing disease (12). More information about the factors that influence Kp colonization and infection is necessary, given the high risk of infection posed to patients colonized by Kp, especially in the context of increasing levels of antibiotic resistance.

Our recent work revealed that the presence of the Kp *ter* operon is associated with bacteremia and pneumonia in colonized patients (13). The experimental interrogation of the *ter* operon using an isogenic mutant revealed that the *ter* operon increases Kp fitness during gut colonization, rather than conferring a fitness advantage during bacteremia and pneumonia (13, 14). In particular, a *terC* mutant is less fit in mice with an increased abundance of bacteria that are known to produce short-chain fatty acids (SCFA). SCFAs, specifically acetate, can inhibit the growth of Kp in a pH-dependent manner (15). However, the exogenous administration of SCFAs to mice during gut colonization, but not *in vitro*, results in a fitness defect that is dependent on the *ter* operon. Therefore, the inhibitory effect of SCFAs is dependent on the presence of specific indigenous gut microbiota (14). This suggests an alternative biological role for the *ter* operon during Kp pathogenesis, wherein this operon is responsive to as yet undefined stresses.

The molecular function of the *ter* operon is cryptic. This operon confers resistance to the oxyanion form of the rare nonessential trace element tellurium, namely, tellurite oxide (TeO_3_^−2^). Additionally, the mechanism by which TeO_3_^−2^ damages bacterial cells is unclear. Tellurium is occasionally grouped with other transition metals that exhibit antibacterial properties; however, as a chalcogen, its toxicity likely differs from those of transition metals. Furthermore, TeO_3_^−2^ is largely absent in the human body and in medical settings (16, 17). Therefore, physiologically relevant stress or stresses other than TeO_3_^−2^ must explain the strong association between the *ter* operon and Kp pathogenesis. The biochemical mechanisms of TeO_3_^−2^-induced stress affect several critical pathways and therefore induce pleiotropic stress (reviewed in [18]), which makes it challenging to infer which physiologically relevant stresses interact with the *ter* operon. The strong oxidizing ability of TeO_3_^−2^ is thought to be primarily responsible for its toxicity, and it has several secondary adverse consequences for the bacterial cell (19). The reduction of TeO_3_^−2^ creates hydroxyl radicals by inhibiting heme biosynthesis (20). As a result, these hydroxyl radicals abrogate DNA synthesis and protein synthesis, exhaust cellular reductases, and oxidize membrane lipids (21–24). In conjunction with the proposed biochemical mechanism of TeO_3_^−2^-induced stress, the *ter* operon may play a role in resistance to colicins (families A, B, and K) and bacteriophages (16). Notably, many of the biochemical mechanisms of TeO_3_^−2^-induced stress are akin to those that Kp may encounter during its pathogenesis. For example, in the gut, where the *ter* operon is conditionally required for complete fitness, colicins are important weapons of bacterial warfare, and host antimicrobial peptides limit pathogen proliferation (25, 26). These stresses kill by attacking the bacterial membrane and DNA, similar to the effects of TeO_3_^−2^ (25, 26). Therefore, exploring the molecular function of the *ter* operon is likely to reveal interesting facets of Kp pathogenesis.

Here, we interrogate the physiologically relevant role of the Kp *ter* operon and associated TeO_3_^−2^-induced stress. First, we identified a novel role for the *ter* operon as a fitness factor during a urinary tract infection (UTI). Concordant with a fitness impact in multiple body sites, we found that the *ter* operon is involved in stress tolerance, a phenotype wherein more tolerogenic cells die slower than less tolerogenic cells in the presence of harmful agents. This phenotype comports with a general, rather than a specific, stress response. Finally, using a systematic approach, we identified novel genes that are associated with TeO_3_^−2^ resistance in Kp lacking the *ter* operon and indeed found that TeO_3_^−2^ acts on diverse biological pathways. This corresponds to a need for a general mechanism of stress response. Collectively, these data suggest a role for the *ter* operon in responding to envelope destabilization, which would thereby result in an enhanced stress tolerance and would potentially explain how *ter* operon function enhances fitness during UTIs and gut colonization.

## RESULTS

### TerC is a bladder fitness factor during urinary tract infections.

**FIG 1:**
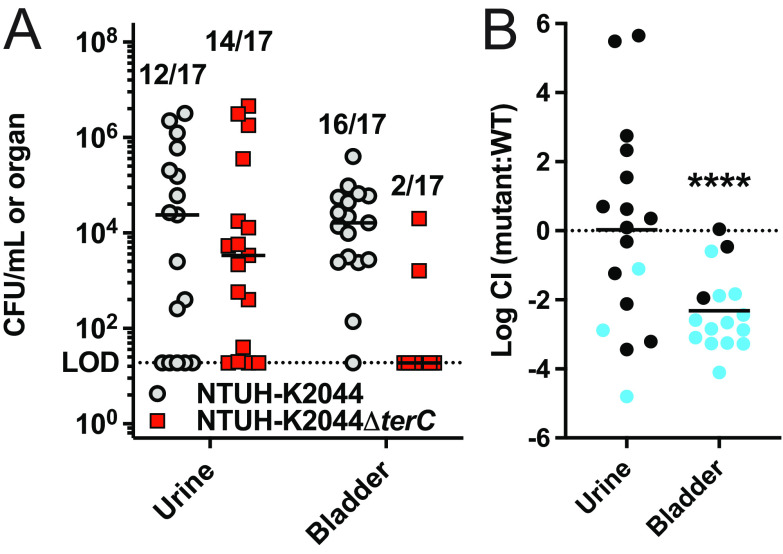
TerC is necessary for complete fitness in the bladder during a urinary tract infection. Mice were transurethrally inoculated with approximately 10^8^ CFU of a 1:1 mix of WT NTUH-K2044 and NTUH-K2044Δ*terC*. (A) The bacterial burden in the urine and bladder was measured after 48 h, and (B) the log_10_ competitive index (CI) of the mutant strain, compared to the WT strain, was calculated for each organ (*N* = 17; mean displayed; ****, *P* < 0.00005; one-sample *t* test). From 20 inoculated mice, CFU were recovered from 17 mice. The numbers above each column in panel A indicate the number of mice (of 17) with detectable CFU. Blue circles had no recoverable NTUH-K2044Δ*terC*. The CI was calculated using the limit of detection CFU value when no CFU were recovered.

Our previous study, in which we identified the strong association between the *ter* operon and infection, was limited to patients who developed pneumonia and bacteremia (13). However, Kp is also an important cause of UTIs in colonized patients (7, 11). In a previous survey of 2,549 urine-associated Kp isolates, we found that 8.4% contained *ter* (14). To test the role of the *ter* operon in a UTI, we competed our *terC* mutant and wild-type (NTUH-K2044) strains 1:1 in a well-established murine transurethral infection model (27). The deletion of *terC* is sufficient to confer susceptibility to K_2_TeO_3_, and it is complemented by *terZ-F* (13, 14). Here, we observed a 2-log fitness defect for the *terC* mutant strain in the bladders of infected mice (Fig. 1). Furthermore, the *terC* mutant strain was mostly absent from the bladders of the infected mice (only 2 out of 17 had detectable colony forming units [CFU]). The observed bladder fitness defect was not due to differences in growth in urine (Fig. S1A–C), and it was not observed *ex vivo* in bladder homogenate (Fig. S1D), indicating that the whole organism, or at least viable tissue, is required in order to observe a TerC-dependent fitness defect. The finding that the *ter* operon is required for complete fitness in the bladder *in vivo* was somewhat surprising, as we have previously demonstrated that TerC is required for complete fitness in the gut in a microbiome-dependent manner (14) and that bladder-indigenous microbiota are either sparse or absent (28). Therefore, these findings suggest a function for the *ter* operon that explains a role relating to fitness in both the gut and bladder.

### TerC is not required for growth on common sugars.

Given the finding that TerC is necessary for complete fitness in two dissimilar body sites, we turned to a hypothesis-driven approach to identify the mechanisms that unify these phenotypes. Structure-based prediction of the function suggested that TerC may act as a sugar transporter (14). Specifically, the highest-confidence functional predictions were hexose:proton symporter activity, galactose transmembrane transporter activity, arabinose transmembrane transporter activity, and fucose transmembrane transporter activity. This activity could explain the fitness defect observed in multiple, unrelated body sites, as perturbations in metabolic flexibility are known to impact Kp site-specific fitness, including that of the gut (29). To test this hypothesis, we compared the growth of the wild-type (WT) and *terC* mutant strains containing an empty pACYC184 vector (NTUH-K2044 pVector and NTUH-K2044Δ*terC* pVector, respectively) and a *terC* complement strain expressing *terZ-F* in *trans* (NTUH-K2044Δ*terC* pTerZ-F) in M9 minimal medium supplemented with various sugars at a final concentration of 0.5%. We previously demonstrated that expression of *terZ-F* is required to complement the K_2_TeO_3_ resistance phenotype (14). TerC was dispensable for growth in the presence of all sugars tested (Fig. S2), which include: the hexoses fucose, galactose, glucose, and rhamnose; the pentoses arabinose and xylose; and the disaccharides lactose and sucrose. The NTUH-K2044ΔterC pTerZ-F strain had small but significant growth defects in lactose, sucrose, and rhamnose, and it grew significantly better than did NTUH-K2044 pVector in galactose, based on an area under the curve (AUC) analysis (Fig. S2). Although not exhaustive, these data indicate that TerC is not required for growth on these eight common sugars.

### TerC is dispensable for resistance to metal-induced and radical oxygen species-induced stress.

Next, we hypothesized that the *ter* operon may be involved in resistance to transition metal-induced stress. Metal-responsive genes are important fitness factors during UTIs (30, 31) and gut colonization (32). Moreover, several transition metal resistance operons are colocalized on *ter* operon-containing plasmids (14), suggesting a conserved role for transition metal resistance for these plasmids. To test this hypothesis, we determined the minimum inhibitory concentration (MIC) of several first-row transition metals that have known functions as micronutrients and are toxic in excess via the induction of redox stress (reviewed in [33] and [34]). As expected, the *terC* mutant strain was more susceptible to K_2_TeO_3_ than was the WT strain, and this phenotype was complemented by expressing *terZ-F* in *trans* (Fig. S3A). No differences in MIC were observed for any transition metals, indicating that the *ter* operon is dispensable for resistance to transition metal-induced stress (Fig. S3A). Moreover, the MIC of K_2_TeO_3_ was substantially lower than that of the tested first-row transition metals, indicating that the biological activity of the first-row transition metals differs from that of K_2_TeO_3_.

As K_2_TeO_3_ is a potent generator of radical oxygen species (ROS), we next explored the hypothesis that the *ter* operon may be involved in resistance to ROS-induced stress (18, 20). Like metal-induced stress, resistance to ROS has been implicated in bacterial fitness during UTIs (35, 36) and gut colonization (37). Moreover, some studies have suggested that the *ter* operon is under transcriptional control by OxyR (38, 39). To test this hypothesis, we determined the impact of TerC on the MIC of three cell-permeable ROS generators: hydrogen peroxide (H_2_O_2_) and the superoxide-generating, redox-cycling compounds paraquat and menadione. As above, TerC was necessary for K_2_TeO_3_ resistance; however, TerC was dispensable for resistance to H_2_O_2_, paraquat, and menadione (Fig. S3B). Collectively, these data suggest an alternative function for the *ter* operon.

### TerC plays a role in stress tolerance.

It is perhaps not surprising that the *ter* operon is dispensable for metal-induced and ROS-induced stress, as there are several factors, such as superoxide dismutase, glutathione, and catalase (reviewed in [40]), that play an important role in detoxifying ROS. Correspondingly, metal and ROS resistance genes are important fitness factors during Kp lung infections (41, 42), in which the *ter* operon is dispensable (13). It may be the case that the *ter* operon plays a different role in resisting TeO_3_^−2^ and the pleiotropic stress that it induces. Therefore, we further considered the structure-function relationship of TerC. While TerC does not appear to be a dedicated sugar transporter, it has been suggested that Ter proteins form a stress-sensing membrane complex that is anchored by TerC (43). A potential role for TerC in sensing or responding to envelope stress was intriguing, as the maintenance of the cell envelope is critical for stress tolerance (44, 45). Notably, TerC is membrane-bound (46).

To explore a role for TerC and the *ter* operon in stress tolerance, we turned to the definitions and guidelines for research on antibiotic persistence (47). These guidelines outline the experimental approach for differentiating stress resistance, tolerance, and persistence. Briefly, resistance is defined as a difference in the MIC of a stress, whereas tolerance and persistence are defined as enhanced survival during stress exposure with no change in MIC. Rather, tolerant cells die more slowly during stress exposure, and persister cells are a small subpopulation of tolerant cells that survive stress exposure better than does the general population. Mechanistically, tolerance is often mediated by specific, population-wide stress responses (reviewed in [48]), whereas persister cells are a stochastically formed subpopulation (reviewed in [49]). These phenotypes can be differentiated by measuring the kill curve in the presence of stress, and they can be summarized as the minimum duration of killing of a predefined percentage of the initial population (MDK_%_). Chemical concentrations of ≥10× MIC are used for these experiments. The differences in stress tolerance between two populations are defined by the differential killing of a large fraction of the initial population, such as differences in the 90% (MDK_90_) or 99% (MDK_99_) MDK values. The difference in persistence between two populations is defined by a biphasic kill curve, wherein initial killing occurs at a similar rate (e.g., identical MDK_90_ or MDK_99_) and then transitions to a differential biphasic state, such as differences in the 99.9% (MDK_99.9_) or 99.99% (MDK_99.99_) MDK values of the initial population.

**FIG 2:**
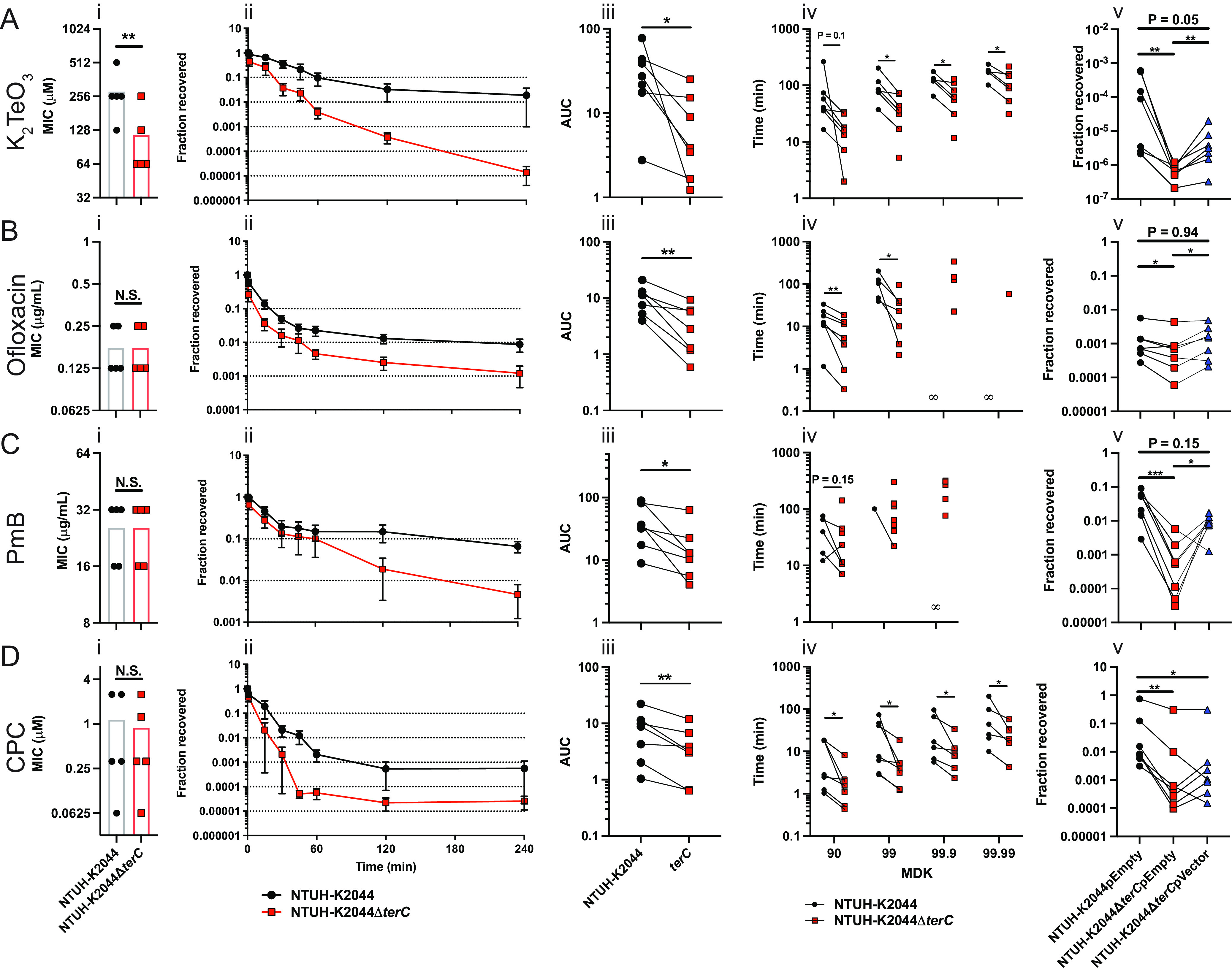
TerC is necessary for tolerance to several stresses. The (A) K_2_TeO_3_ MIC (i: *n* = 5 independent experiments; median displayed; **, *P* < 0.005; ratio paired *t* test) was calculated for the NTUH-K2044 and NTUH-K2044Δ*terC* strains using broth microdilution. Kill curves (ii: *n* = 7 independent experiments; mean displayed ± SEM) were generated for these strains via the addition of a standard K_2_TeO_3_ concentration of 1 mM to overnight cultures. The (iii) AUC and (iv) MDK were calculated from these kill curves (*n* = 7 per group; *, *P* < 0.05; **, *P* < 0.005; ratio paired *t* test; ∞ indicates an incalculable MDK). Finally, the NTUH-K2044 pVector, NTUH-K2044Δ*terC* pVector, and NTUH-K2044Δ*terC* pTerZ-F survival was assessed at 4 h (240 min) post 1 mM K_2_TeO_3_ exposure (v: *n* = 6 to 7 independent experiments; median displayed; *, *P* < 0.05; **, *P* < 0.005; ***, *P* < 0.0005; one-way ANOVA followed by Tukey’s multiple-comparison test). These experiments were repeated for ofloxacin (B), polymyxin B (PmB) (C), and cetylpyridinium chloride (CPC) (D). The standard concentrations for the killing assays for ofloxacin, polymyxin B, and cetylpyridinium chloride were 250 μg/mL, 500 μg/mL, and 25 μM, respectively. For subpanels iii, iv, and v, the connecting lines indicate paired biological replicates.

Using these guidelines, we assessed the role of TerC in stress tolerance. First, we characterized the dynamics of K_2_TeO_3_-mediated killing and observed the more rapid killing of the *terC* mutant, compared to its parent strain (Fig. 2, panels Ai and Aii), which was determined to be significantly different, based on an AUC analysis (Fig. 2, panel Aiii) and on the interpolation of exact MDK values (Fig. 2, panel Aiv). Finally, TerC-dependent survival at 4 h post stress exposure was complemented in *trans* (Fig. 2, panel Av). Next, we explored the killing dynamics in response to ofloxacin-induced stress. Fluoroquinolones, such as ofloxacin, kill bacterial cells through the inhibition of DNA gyrase and are commonly used to generate persister cells in a laboratory setting (50). The MIC of ofloxacin was identical between the *terC* mutant and its parent strain (Fig. 2, panel Bi), indicating that TerC is not an ofloxacin resistance factor. Interestingly, we observed significant differences in the kill curves between these two strains (Fig. 2, panels Bii and iii). The interpolation of MDK values revealed a significant difference between the MDK_90_ and MDK_99_ values of the *terC* mutant and its parent (Fig. 2, panel Biv), indicating that the wild-type strain was significantly more tolerant to ofloxacin-induced stress than was the *terC* mutant. The MDK_99.9_ and MDK_99.99_ were incalculable for the wild-type strain, as ofloxacin failed to kill this proportion of cells. As described above, the TerC-dependent survival at 4 h post stress exposure was complemented in *trans* (Fig. 2, panel Bv). Collectively, these data suggest that TerC may be necessary for ofloxacin tolerance.

To investigate this tolerance phenotype further, we tested a second antibiotic, polymyxin B (PmB), which has a different mechanism of action than does ofloxacin, namely, disruption of the bacterial cell envelope. As was observed with ofloxacin, the MIC of PmB was identical for both the *terC* mutant and its parent strain (Fig. 2. Ci). Notably, we observed significant differences in the kill curves between these two strains in response to PmB-induced stress, wherein the wild-type strain was killed more slowly than was the *terC* mutant (Fig. 2, panels Cii and iii). The MDK_90_ of the *terC* mutant trended lower than its parent strain, and the MDK_99_ and MDK_99.9_ values were incalculable for the wild-type strain (Fig. 2, panel Ci). Complementation in *trans* restored survival at 4 h post stress exposure (Fig. 2, panel Cv). As with ofloxacin, these data indicate that the *terC* mutant is less tolerant to PmB-induced stress than is its parent strain. Finally, we repeated these experiments with cetylpyridinium chloride (CPC), a quaternary ammonium compound that disrupts the cell envelope and leads to the leakage of intracellular contents and death (51). As with PmB, no differences were observed between the MIC of the wild-type strain and that of the *terC* mutant (Fig. 2, panel Di); however, the *terC* mutant was killed more rapidly than was its parent strain due to CPC-induced stress, which was reflected in all of the MDK values (Fig. 2, panels Dii–iv). Finally, 4 h post stress exposure, survival was complemented in *trans* (Fig. 2, panel Dv). Collectively, these data indicate that TerC is necessary for tolerance to stresses with distinct mechanisms of action and potentially explains the requirement of TerC for complete fitness in the gut and bladder.

### A systematic screen reveals diverse genes associated with K_2_TeO_3_ resistance.

**FIG 3:**
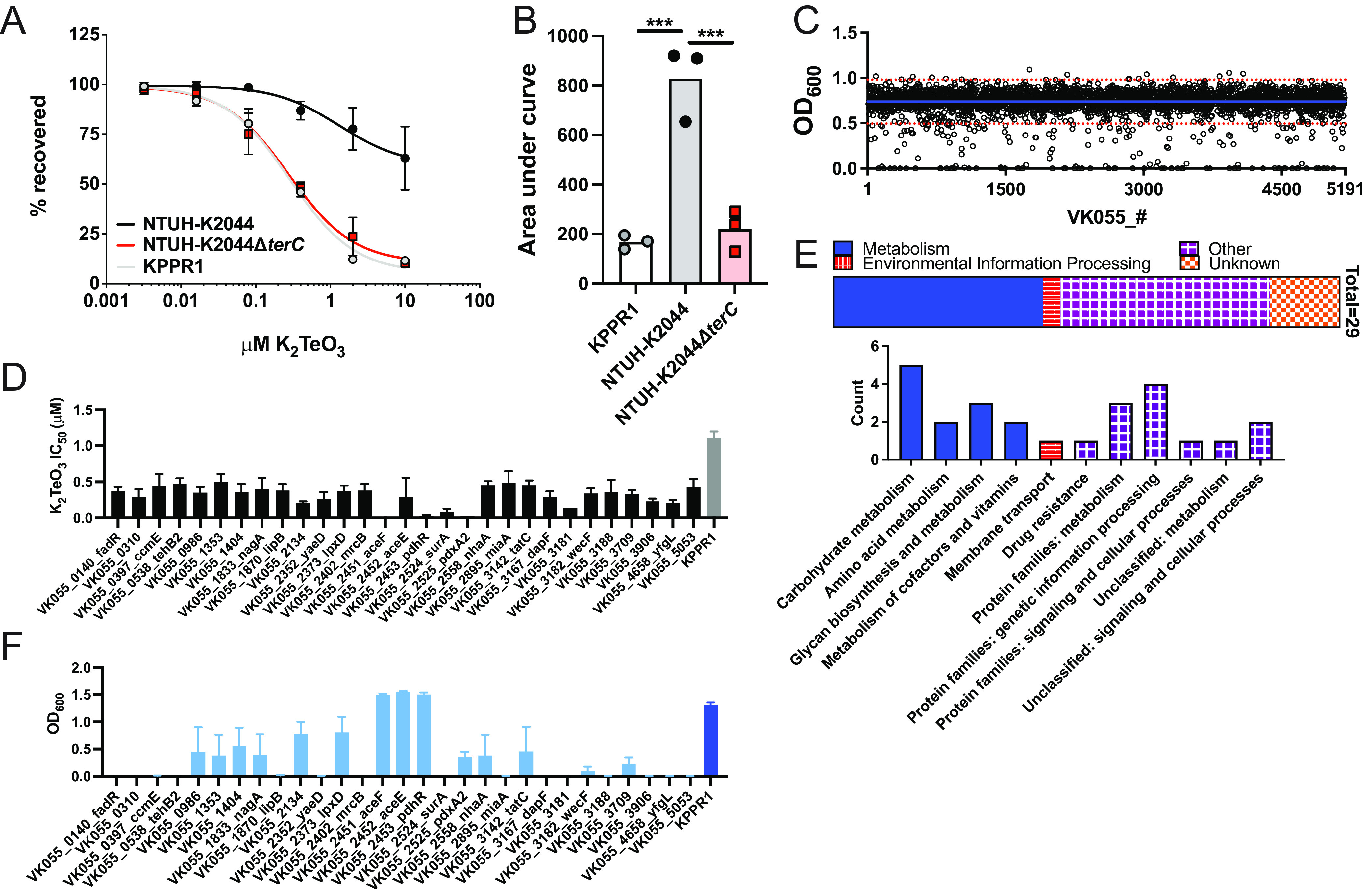
Systematic screen of K_2_TeO_3_ resistance. The KPPR1 strain, which lacks the *ter* operon, as well as the the NTUH-K2044 and NTUH-K2044Δ*terC* strains were cultured in increasing concentrations of K_2_TeO_3_ (A). The area under the curve (AUC) was calculated from these dose-response curves (B) (mean displayed ± SEM; ****, *P* < 0.0005; one-way ANOVA followed by Tukey’s multiple-comparison test). (C) 3,733 individual Tn insertion mutants were cultured in the presence of 1 μM K_2_TeO_3_ and were measured at OD_600_. The blue line is the mean OD_600_, and the red lines are ± 2 SD from the mean. Each symbol is an individual mutant, ordered by its gene number (VK055_#). (D) Exact K_2_TeO_3_ IC_50_ values of validated Tn insertion mutants (*n* = 3 to 5 independent experiments). (E) KEGG BRITE functional hierarchies were assigned for the validated Tn insertion mutants. The highest order hierarchies are shown in the horizontal stacked bar chart, and the second-highest order hierarchies are shown in the unstacked bar chart. (F) The validated Tn insertion mutants were cultured in the presence of 0.5 μg/mL PmB (*n* = 3 independent experiments).

The finding that TerC is required for tolerance, but not resistance, to stresses with differing mechanisms of action comports with its requirement for resistance to the pleiotropic stress induced by K_2_TeO_3_; however, this finding does not implicate a specific pathway or a cellular compartment of action for the *ter* operon. To determine whether K_2_TeO_3_ acts on a specific pathway or cellular compartment, we turned to a systematic approach to identify the genes and pathways required to mitigate the effects of the K_2_TeO_3_-induced stress when the *ter* operon was absent. To this end, we employed an ordered, condensed transposon (Tn) library that was constructed in the Kp strain KPPR1, does not encode the *ter* operon, and is susceptible to K_2_TeO_3_ (Fig. 3A and B). This Tn library contains individual insertions in 3,733 genes, covering 72% of all of the available open reading frames in the genome (52). First, we determined the concentration at which KPPR1 growth is partially inhibited by K_2_TeO_3_ to be approximately 1 μM. We aimed to screen our Tn library at this concentration to identify mutants conferring both resistance (increase in growth) and susceptibility (decrease in growth) to K_2_TeO_3_. Then, we cultured each mutant in the presence of 1 μM K_2_TeO_3_ and assessed its growth by measuring the growth at OD_600_ (Fig. 3C). If the growth of a mutant was two standard deviations above or below the mean growth of all mutants, we categorized the interrupted gene as potentially being important for K_2_TeO_3_ resistance. Initially, we found 129 candidate susceptible mutants and 15 candidate resistant mutants (Fig. 3C). It was not surprising that we identified more susceptible than resistant mutants, as previous studies using other bacteria to identify the genes involved in K_2_TeO_3_ resistance required several passages in the presence of K_2_TeO_3_ to identify such loci (20). As our screen was performed at a standard concentration of K_2_TeO_3_, we next aimed to validate our candidate mutants. We preliminarily excluded 14 mutants due to an observed growth defect in Luria-Bertani Broth (LB) in the absence of K_2_TeO_3_. Then, we determined the concentration of K_2_TeO_3_ that inhibited 50% of the bacterial growth (IC_50_) for our remaining candidates. Of the 138 candidates, 79 had a K_2_TeO_3_ IC_50_ value that corroborated our original screen, and 29 had a significantly lower (*q* ≤ 0.1) IC_50_ value than that of the parent strain (Table 1; Fig. 3D; Table S1). To further characterize the genes associated with K_2_TeO_3_ resistance, we assessed the gene function using the Kyoto Encyclopedia of Genes and Genomes (KEGG) (Fig. 3E; Table S1). “Metabolism” (*n* = 12 out of 29) and “Other” (*n* = 12 out of 29) were the most well-represented categories. The assignment of more granular functions of these genes revealed that the most common function was carbohydrate metabolism (*n* = 5 out of 29), and this was followed by genetic information processing (*n* = 4 out of 29). These findings suggest that many diverse genes are necessary for growth in the presence of K_2_TeO_3_, rather than a set of related genes with conserved functions.

**TABLE 1:** Insertion mutants involved in K_2_TeO_3_ resistance^*a*^

Gene name	Annotation	Subcellular location	K2TeO3 IC50 ± SEM (KPPR1 = 1.11 ± 0.09)	*P* value	*q* value
*VK055_0140_fadR*	fatty acid metabolism transcriptional regulator FadR	cytoplasm	0.37 ± 0.06	0.003060	0.010447
*VK055_0310*	bacterial regulatory helix-turn-helix, LysR family protein		0.29 ± 0.11	0.000093	0.000656
*VK055_0397_ccmE*	cytochrome c-type biogenesis protein CcmE	integral component of membrane	0.44 ± 0.17	0.002301	0.009113
*VK055_0538_tehB2*	tellurite resistance protein TehB	cytoplasm	0.47 ± 0.08	0.006082	0.019425
*VK055_0986*	hypothetical protein	NA	0.35 ± 0.08	0.001477	0.006521
*VK055_1353*	putative l,d-transpeptidase YcfS	periplasmic space	0.50 ± 0.11	0.027590	0.068284
*VK055_1404*	periplasmic glucans biosynthesis protein MdoG	periplasmic space	0.36 ± 0.11	0.001024	0.005068
*VK055_1833_nagA*	N-acetylglucosamine-6-phosphate deacetylase NagA	cytoplasm	0.40 ± 0.16	0.002208	0.009106
*VK055_1870_lipB*	lipoyl(octanoyl) transferase LipB	cytoplasm	0.38 ± 0.09	0.002789	0.009860
*VK055_2134*	putative dTDP-glucose pyrophosphorylase		0.21 ± 0.02	0.000011	0.000120
*VK055_2352_yaeD*	d,d-heptose 1,7-bisphosphate phosphatase GmhB	cytoplasm	0.26 ± 0.10	0.000020	0.000176
*VK055_2373_lpxD*	UDP-3-O-[3-hydroxymyristoyl] glucosamine N-acyltransferase LpxD	cytoplasm	0.37 ± 0.08	0.002434	0.009268
*VK055_2402_mrcB*	penicillin-binding protein 1B MrcB	peptidoglycan-based cell wall	0.38 ± 0.09	0.002789	0.009860
*VK055_2451_aceF*	pyruvate dehydrogenase E2 component AceF	cytoplasm	0.01 ± 0.00	0.000001	0.000001
*VK055_2452_aceE*	pyruvate dehydrogenase E1 component AceE		0.29 ± 0.27	0.000001	0.000001
*VK055_2453_pdhR*	transcriptional repressor for pyruvate dehydrogenase complex PdhR	cytoplasm	0.03 ± 0.01	0.000001	0.000001
*VK055_2524_surA*	peptidyl-prolyl cis-trans isomerase SurA	periplasmic space	0.08 ± 0.05	0.000001	0.000001
*VK055_2525_pdxA2*	4-hydroxythreonine-4-phosphate dehydrogenase PdxA2	cytoplasm	0.02 ± 0.00	0.000001	0.000001
*VK055_2558_nhaA*	na+/H+ antiporter NhaA	integral component of membrane	0.45 ± 0.06	0.015340	0.039965
*VK055_2895_miaA*	tRNA dimethylallyltransferase MiaA	cytoplasm	0.49 ± 0.16	0.013757	0.038914
*VK055_3142_tatC*	sec-independent protein translocase protein TatC	integral component of membrane	0.45 ± 0.07	0.014802	0.039606
*VK055_3167_dapF*	diaminopimelate epimerase DapF	cytoplasm	0.29 ± 0.08	0.000148	0.000977
*VK055_3181*	enterobacterial common antigen polymerase WzyE	integral component of membrane	0.14 ± 0.00	0.000001	0.000002
*VK055_3182_wecF*	dTDP-N-acetylfucosamine:lipid II N-acetylfucosaminyltransferase WecF	integral component of membrane	0.34 ± 0.07	0.001236	0.005826
*VK055_3188*	UDP-N-acetyl-d-mannosaminuronic acid dehydrogenase WecC		0.36 ± 0.17	0.000323	0.001880
*VK055_3709*	shikimate kinase AroK	cytoplasm	0.33 ± 0.06	0.000970	0.005055
*VK055_3906*	hypothetical protein	NA	0.23 ± 0.04	0.000021	0.000177
*VK055_4658_yfgL*	outer membrane protein assembly factor BamB	integral component of membrane	0.21 ± 0.04	0.000007	0.000088
*VK055_5053*	DNA gyrase inhibitor SbmC	cytoplasm	0.43 ± 0.11	0.008174	0.025287

*^a^*Empty cells indicate that no subcellular location was predicted.

To determine whether the genes involved in K_2_TeO_3_ resistance are also involved in the resistance against stressors for which TerC was required for tolerance, we screened the mutants validated for increased K_2_TeO_3_ sensitivity for growth in the presence of PmB. Interestingly most genes involved in K_2_TeO_3_ resistance were also required for growth in the presence of PmB (Fig. 3F). The pyruvate dehydrogenase complex (*aceF*, *aceE*, *pdhR*) was one notable exception to this finding. Given that PmB is a membrane-active antibiotic, this finding further supports the indication that K_2_TeO_3_ destabilizes the Kp envelope. Moreover, this suggests that the function of the *ter* operon is to aid in envelope stabilization or to respond to envelope destabilization, which leads to enhanced stress tolerance and enhanced fitness in the gut and bladder.

A gene ontology biological process enrichment analysis revealed a single enriched pathway among these individual genes: enterobacterial common antigen (ECA) biosynthetic process (42.23-fold enrichment, false discovery rate [FDR] *P* value = 5.25 × 10^−2^). Notably, the additional ECA genes *wecG* and *wecA* narrowly missed our validation criteria (Table S1). This finding supports a potential role for the *ter* operon in maintaining envelope stability or in responding to envelope destabilization, which comports with a role in stress tolerance.

### K_2_TeO_3_ disrupts the Kp cell envelope.

**FIG 4:**
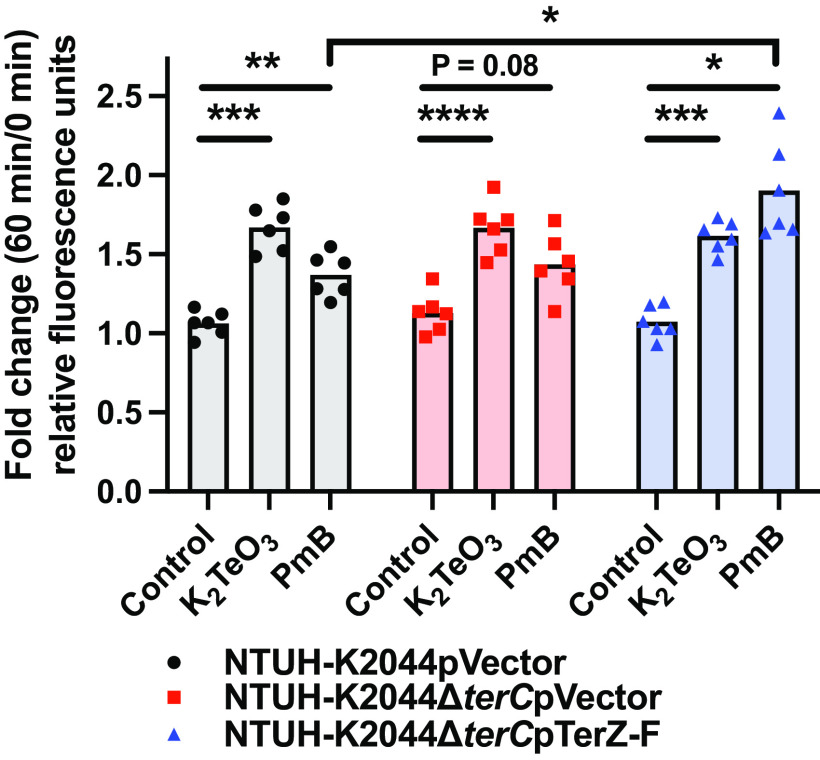
EtBr accumulates in K_2_TeO_3_ and polymyxin B treated Kp. Stationary-phase NTUH-K2044 pVector, NTUH-K2044ΔterC pVector, and NTUH-K2044ΔterC pTerZ-F were exposed to 1 mM K_2_TeO_3_ or 500 μg/mL PmB. EtBr accumulation (fluorescence) was measured at the baseline and at 1 h postexposure (*n* = 6 independent experiments; mean displayed; *, *P* < 0.05; **, *P* < 0.005; ***, *P* < 0.0005; one-way ANOVA followed by Tukey’s multiple-comparison test).

Given the finding that many genes associated with K_2_TeO_3_ resistance in a strain lacking *ter* may play a role in maintaining envelope stability, we next aimed to determine whether K_2_TeO_3_ disrupts the cell envelope and whether the *ter* operon stabilizes the envelope. Fluorescence-based ethidium bromide (EtBr) accumulation assays can be used to assess envelope damage (53, 54), wherein EtBr accumulates in the periplasm or intercalates in the cellular DNA following cell envelope disruption. As colistin has been shown to disrupt both the outer and inner membranes of the Gram-negative envelope (55), we used PmB as a positive control. Following exposure to K_2_TeO_3_ and PmB at the same concentrations used in the killing assays (Fig. 2), Kp displayed higher levels of EtBr accumulation than did the no treatment controls (Fig. 4). This phenotype was independent of TerC, suggesting that the effects of *ter* are downstream of the initial disruption of the cell envelope (Fig. 4). These data demonstrate that K_2_TeO_3_ disrupts the cell envelope and, in conjunction with the data in Fig. 2, panel Aii (difference in fraction recovered at 60 min post K_2_TeO_3_ exposure), suggest that the effects of *ter* are downstream of envelope destabilization.

## DISCUSSION

The work presented here advances our understanding of the physiological role of the Kp *ter* operon. Our results indicate that a physiological role of the *ter* operon is to respond to envelope stress during the colonization and infection of specific body sites to tolerate these stressful environments and enhance fitness. These effects are likely downstream of the initial insult to the envelope. Previously, we have shown that this cryptic operon is highly associated with Kp pneumonia and bacteremia in colonized patients (13), and further work demonstrated that this association was due to a TerC-dependent fitness advantage that is conferred in the gut (14). Here, we demonstrate a novel role for TerC as a fitness factor during a urinary tract infection. The identification of TerC as a bladder fitness factor using a hypervirulent Kp strain (NTUH-K2044) is noteworthy, as several recent reports have indicated that hvKp are an important cause of asymptomatic bacteriuria and UTIs (56–60). As hypervirulent strains become more prevalent and hypervirulent, antibiotic resistant strains emerge, and it is critical to identify compartment-specific fitness factors (61, 62). Additionally, this finding that TerC is a fitness factor during a urinary tract infection implies a conserved mechanism of fitness enhancement in the gut and bladder that is dispensable in the lung and blood (13, 14).

To identify this conserved mechanism, we first focused on the known mechanisms of K_2_TeO_3_-induced stress that functionally overlap gut and bladder fitness factors. We determined that TerC is dispensable for metal resistance and ROS resistance. We also determined that TerC is dispensable for the transport of several common sugars. Rather, we identified a novel role for TerC in stress tolerance, wherein Kp lacking TerC were killed more rapidly in the presence of several stresses. Given the pleiotropic effects of K_2_TeO_3_ on the bacterial cell, a role for the *ter* operon during a general stress response is appealing. This could explain how the *ter* operon enhances fitness in biologically distinct sites, at which the specific stresses Kp encounters are likely to differ. To identify a specific function for the *ter* operon, we undertook a systematic screen of K_2_TeO_3_ resistance in a Kp strain that lacked the *ter* operon. This screen did not reveal a consensus molecular function or a specific cellular compartment of action, but it did suggest that K_2_TeO_3_-induced stress is primarily experienced at the cell envelope. Several of these genes were also required for growth in the presence of PmB, which is a membrane-active antibiotic. This finding affirms the necessity for TerC during stress tolerance. Finally, we demonstrate that K_2_TeO_3_ disrupts the cell envelope, though these effects were independent of *ter*, which suggests an indirect means of responding to envelope stress.

Tolerance has received significant attention due to its contribution to antibiotic treatment failure and to the development of antibiotic resistance; however, this neglects the role of tolerance to other stresses that are induced by factors that are not characterized as classical antibiotics, such as bacteriocins or detergents. This is especially relevant in the context of PmB. Polymyxins bind to LPS in the outer and cytoplasmic membrane, resulting in cell lysis and death (55). The finding that TerC is necessary for tolerance to PmB supports the assertion proposed in other studies that the Ter proteins form a stress-sensing membrane complex that may influence transmembrane permeability (43). Similarly, the quaternary ammonium compound CPC kills bacterial cells through its integration into and disruption of the cell envelope. The biological relationship between TerC and ofloxacin tolerance is less clear, though the membrane peptide TisB enhances the tolerance to ciprofloxacin through the disruption of the proton motive force (63). Additionally, several mechanistically divergent antibiotics, including fluoroquinolones, induce inner membrane damage and cytoplasmic condensation, which leads to bacterial cell death (64). Therefore, the destabilization of the cell envelope may have secondary effects that impact tolerance. The exploration of TerC-dependent tolerances to other diverse stresses is likely to further refine our understanding of the biology of the *ter* operon.

Our systematic screen of K_2_TeO_3_ resistance revealed both expected and novel loci that are associated with K_2_TeO_3_ resistance (Table 1). The interruption of the pyruvate dehydrogenase complex, which resulted in increased K_2_TeO_3_ susceptibility, was expected and serves as a validation of the approach, as the heterologous expression of *aceE and aceF* leads to enhanced K_2_TeO_3_ resistance (65). Commensurately, TehB is a known K_2_TeO_3_ resistance protein that, in conjunction with TehA, confers resistance through the volatilization of tellurite through methyltransferase activity (18). An insertion mutant in *tehA* was not present in our Tn library. The finding that the ECA synthesis locus is a significant contributor to K_2_TeO_3_ resistance is particularly intriguing, as this conserved locus has been implicated in many critical facets of the biology of Enterobacterales. ECA is a carbohydrate structure that is characteristically found in the bacterial envelope, where it is attached to lipopolysaccharides (LPS) and peptidoglycan (reviewed in [66]). A disruption of ECA in Kp reduces virulence in murine pneumonia and bacteremia models, though this phenotype is largely dependent on the stability of the LPS, rather than ECA itself (67). Regardless of whether the observed K_2_TeO_3_ resistance phenotype is dependent on LPS stability, the envelope appears to be a critical mediator of K_2_TeO_3_ resistance for Kp, independent of *ter*. A role for envelope stability in K_2_TeO_3_ resistance is also supported by increased K_2_TeO_3_ sensitivity of the *bamB*, *gmhB*, and *mdoG* mutants. The Bam complex (BamABCDE) is responsible for the proper insertion of proteins into the outer membrane (reviewed in [68]), whereas GmhB plays a role in LPS biosynthesis (69, 70) and MdoGH is critical for the biosynthesis of osmoregulated periplasmic glucans (reviewed in [71]). Therefore, the disruption of these genes or the ECA biosynthesis locus may result in a destabilized envelope.

Although this study revealed novel aspects of the biology of the *ter* operon, it is not without its limitations. First, this study provides insight into the function of the *ter* operon, but the molecular mechanisms underlying its function remain unknown. Second, the use of a transposon library that is comprised of single-gene insertions may limit the ability to identify every gene involved in K_2_TeO_3_ resistance. Some of these insertions may not sufficiently disrupt gene function to the same degree as could alternative insertions that are present in a more complex library. Finally, the experiments in this study were limited to two strains: the *ter* operon containing strain NTUH-K2044 and the *ter* operon lacking strain KPPR1. The use of these strains is convenient due to available molecular tools and because they are well-characterized; however, both strains are hypervirulent stains. Therefore, they do not represent the complete genomic breadth of Kp. While the *ter* operon is highly associated with hypervirulent Kp, it is not limited to hypervirulent strains (14). Interestingly, an analogous study using a murine UTI model reported similar bladder bacterial loads using a nonhypervirulent strain (72). Future studies dissecting the function of the *ter* operon should consider both hypervirulent and nonhypervirulent Kp strains. Despite these limitations, this study represents a significant advancement in our understanding of the role of the *ter* operon during Kp pathogenesis.

## MATERIALS AND METHODS

### Ethics statement.

The human sample collection was approved by and performed in accordance with the Institutional Review Boards (IRB) of the University of Michigan Medical School (study number HUM00004949). The animal studies were performed in strict accordance with the recommendations in the Guide for the Care and Use of Laboratory Animals (102). The University of Michigan Institutional Animal Care and Use Committee approved this research (PRO00009173).

### Materials, media, and bacterial strains.

All materials and chemicals were purchased from Sigma-Aldrich (St. Louis, MO) or Fisher Scientific (Hampton, NH) unless otherwise noted. The construction and validation of the isogenic *terC* mutant, the pTerZ-F complementation plasmid, the empty vector, and the complemented strain are described elsewhere (13, 14). All of the strains were grown in the presence of the appropriate antibiotics for all experiments.

### Murine UTI model.

The ascending UTI model that was used in this study has been described elsewhere (27, 73, 74). Briefly, the NTUH-K2044 and NTUH-K2044Δ*terC* strains were cultured overnight from single colonies in LB at 37°C. After overnight growth, the strains were mixed 1:1 and adjusted to a final concentration of 2× 10^9^ CFU/mL in sterile phosphate-buffered saline (PBS). An aliquot of this inoculum was plated on LB agar containing the appropriate antibiotics. The plates were incubated overnight at 27°C to enumerate the input CFU and the exact ratio. Then, the mice were anesthetized with a weight-appropriate dose (0.1 mL for a mouse weighing 20 g) of ketamine-xylazine (80 to 120 mg/kg ketamine and 5 to 10 mg/kg xylazine) via intraperitoneal injection. 50 μL of inoculum was administered transurethrally into male CBA/J mice over a 30 s period to deliver 10^8^ CFU per mouse. After 48 h, the urine was collected, the mice were euthanized via inhalant anesthetic overdose, and the bladder was collected in sterile PBS. The bladders were homogenized, and all of the samples were plated on LB agar containing the appropriate antibiotics using an Autoplate 4000 (Spiral Biotech, Norwood, MA) and incubated overnight at 27°C to enumerate the CFU. The bladder homogenates for the growth assays were prepared from uninfected mice. The bladders were collected into 1 mL sterile PBS, homogenized, and then centrifuged at 21,130 × *g* for 5 min at 4°C to pellet the contaminating bacteria. Then, the supernatant was stored at −80°C until use.

### Human urine collection.

Human urine was collected from women (ages 21 to 40) from whom informed consent had been obtained, who had no symptoms of UTI or bacteriuria, and who had not taken antibiotics in the prior 2 weeks. Deidentified samples from least 4 volunteers were pooled and filter sterilized using a 0.22-μm filter (MilliporeSigma, Burlington, MA), as previously described (74).

### Growth assays.

The NTUH-K2044 pVector, NTUH-K2044Δ*terC* pVector, and NTUH-K2044Δ*terC* pTerZ-F strains were cultured overnight from single colonies in M9 minimal medium containing 0.5% glucose (M9-Glu), which was then diluted to an OD_600_ of 0.01 in M9 minimal medium containing 0.4% or 0.5% arabinose, fucose, galactose, glucose, lactose, rhamnose, sucrose, xylose, 100% human urine, or 100% murine bladder homogenate. 100 μL of this subculture were plated into a single well of a U bottom 96-well plate in triplicate. Then, that plate was sealed using optical adhesive film (Applied Biosystems, Waltham, MA). This plate was incubated at 37°C with aeration, and OD_600_ readings were taken every 15 min using an Eon microplate reader with Gen5 software (Version 2.0, BioTek, Winooski, VT) for 24 h. The area under the curve was quantified using Prism 8 (GraphPad Software, La Jolla, CA).

### MIC determination.

The NTUH-K2044, NTUH-K2044Δ*terC*, NTUH-K2044 pVector, NTUH-K2044Δ*terC* pVector, and NTUH-K2044Δ*terC* pTerZ-F strains were cultured overnight from single colonies in M9-Glu. Overnight cultures were diluted to 10^7^ CFU/mL into M9-Glu with a 2× concentration of strain-appropriate antibiotics. Then, metals, ROS generators, antibiotics, biocides, or K_2_TeO_3_ was diluted in M9-Glu to a 2× final concentration. 100 μL of this solution were plated into a single well of a U bottom 96-well plate in triplicate and then 2-fold serially diluted into M9-Glu 10 times, discarding 50 μL of the last dilution to achieve a final volume of 50 μL in each well, leaving the last well as only medium. Finally, 50 μL of the 2× culture dilution were plated across each serial dilution to achieve a final cell density of 5× 10^6^ CFU/mL. This plate was sealed using optical adhesive film (Applied Biosystems, Waltham, MA) and incubated at 37°C for 24 h. After 24 h, the MIC of each compound was defined as the lowest concentration that fully inhibited bacterial growth. Due to the opacity of the high concentration metal solutions, the MICs of the metals were confirmed via replicate plating onto LB-agar and overnight culture at 37°C.

### Killing assays.

The NTUH-K2044, NTUH-K2044Δ*terC*, NTUH-K2044 pVector, NTUH-K2044Δ*terC* pVector, and NTUH-K2044Δ*terC* pTerZ-F strains were cultured overnight from single colonies in M9-Glu. To ensure culture uniformity, overnight cultures were diluted 1:1,000 into fresh M9-Glu. Following overnight growth, 500 μL of culture were removed, and the cells were pelleted at 10,000 × *g* for 3 min, washed once in 500 μL sterile PBS, resuspended in 500 μL sterile PBS, and serial plated onto LB-agar containing the appropriate antibiotics to determine the initial cell density. Then, K_2_TeO_3_, ofloxacin, polymyxin B, and cetylpyridinium chloride were added to these cultures to a final concentration of 1 mM, 250 μg/mL, 500 μg/mL, and 25 μM, respectively, from stocks prepared in M9-Glu. 500 μL of culture were removed at the indicated time points, and the cells were processed as described above. All of the plates were incubated overnight at 27°C, and the bacterial CFU/mL was quantified after overnight growth. Bacterial killing was summarized as the fraction of bacterial cells recovered at each time point, wherein the CFU/mL at a given time point was normalized to the initial CFU/mL. The area under the curve was calculated, and the minimum duration killing value was interpolated from kill curves following the log transformation of the fraction recovered values using Prism 8 (GraphPad Software, La Jolla, CA).

### Tn library screen.

The construction, ordering, and condensation of the KPPR1 Tn library has been described elsewhere (41, 52). This arrayed library was cultured overnight in flat bottom 96-well plates at 37°C in LB containing 40 μg/mL kanamycin. After overnight growth, arrayed Tn insertion mutants were subcultured into U bottom 96-well plates of LB containing 40 μg/mL kanamycin and 1 μM K_2_TeO_3_, and they were cultured overnight at 37°C. The bacterial growth after 24 h was measured at OD_600_ using an Eon microplate reader with Gen5 software (Version 2.0, BioTek, Winooski, VT). This assay was independently repeated twice to achieve three replicates of K_2_TeO_3_ growth. The candidate genes involved in K_2_TeO_3_ resistance were those for which the mean growth in the presence of 1 μM K_2_TeO_3_ was two standard deviations above (mean OD_600_ = 0.980) or below (mean OD_600_ = 0.495) the mean of the growth values of all of the strains (mean OD_600_ = 0.738). The candidate insertion mutants and the parent strain KPPR1 were then cultured overnight from single colonies in LB containing the appropriate antibiotics. Overnight cultures were diluted to 10^7^ CFU/mL into LB with a 2× concentration of the appropriate antibiotics. Then, K_2_TeO_3_ was diluted in LB to a 2× final concentration. A serial dilution was performed as described above (see “MIC determination”), except the 2× K_2_TeO_3_ solution was serially diluted 6 times instead of 10. The 2× culture dilution was then plated across each serial dilution, and the plate was sealed using optical adhesive film (Applied Biosystems, Waltham, MA) and incubated at 37°C for 24 h. The bacterial growth was measured at OD_600_ using an Eon microplate reader with Gen5 software (Version 2.0, BioTek, Winooski, VT), and the exact IC_50_ values were interpolated using a sigmoidal four-parameter logistic curve using Prism 8 (GraphPad Software, La Jolla, CA). This was repeated three to five times per candidate insertion mutant. A candidate insertion mutant was considered validated if its mean exact IC_50_ value was higher or lower than that of the parent KPPR1 strain that corresponded to its original screen results.

To further characterize the validated insertion mutations, the gene names, annotations, and BRITE functional hierarchies were assigned using the Kyoto Encyclopedia of Genes and Genomes (75, 76), using the VK055 gene number as the search criterion. A cellular compartment was assigned using the gene ontology terms in UniProt (77). A GO enrichment analysis was performed using the PANTHER Overrepresentation Test (release 2021-02-24) Escherichia coli as the reference list (78). For the validation experiments, the insertion mutants and the parent strain KPPR1 were cultured at 37°C in LB broth containing the appropriate antibiotics, and they were arrayed into flat bottom 96-well plates in triplicate. Then, the arrayed insertion mutants were diluted 1:100 into LB broth containing the appropriate antibiotics and 0.5 μg/mL polymyxin B in U bottom 96-well plates. The plates were sealed with optical adhesive film (Applied Biosystems, Waltham, MA) and incubated at 27°C for 24 h. After 24 h, the bacterial growth was measured at OD_600_ using an Eon microplate reader with Gen5 software (Version 2.0, BioTek, Winooski, VT). This assay was repeated twice more to achieve three replicates.

### Ethidium bromide accumulation assay.

The NTUH-K2044 pVector, NTUH-K2044Δ*terC* pVector, and NTUH-K2044Δ*terC* pTerZ-F strains were cultured overnight from single colonies in M9-Glu. Following overnight growth, approximately 2× 10^9^ CFU were harvested via centrifugation, resuspended in 2 mL of sterile PBS with or without 1 mM K_2_TeO_3_ or 500 μg/mL polymyxin B, and incubated at 37°C with shaking at 225 rpm. 1 mL of cells was immediately removed, harvested by centrifugation, resuspended in 1 mL of PBS containing 10 μM ethidium bromide, and incubated at room temperature in the dark for 10 min. Following the incubation, the fluorescence was measured in black-walled, clear-bottomed 96-well plates using an excitation of 510 nm and emission of 600 nm, using a Synergy H1 Hybrid Multi-Mode microplate reader with Gen5 software (Version 2.0, BioTek, Winooski, VT). The bacterial density was measured in tandem at OD_600_. This procedure was repeated after 60 min of exposure to PBS with or without K_2_TeO_3_ or polymyxin B. The fluorescence was normalized to the bacterial density, and then the fold change in relative fluorescent units was determined by dividing the normalized relative fluorescent units at 60 min by the normalized relative fluorescent units at 0 min.

### Statistical analysis.

For the *in vitro* studies, all of the experimental replicates represent biological replicates performed on different days. For the statistical analysis, the experimental values were log-transformed, and a two-tailed ratio paired *t* test or a repeated measures one-way ANOVA followed by Tukey’s multiple-comparison test was used to determine the statistical significance of the differences between the groups. For the *in vivo* studies, all of the experiments were repeated twice with independent bacterial cultures. Following the CFU quantification, competitive indices ([CFU mutant output / CFU WT output] / [CFU mutant input / CFU WT input]) were calculated and then log-transformed. A one-sample *t* test compared to a hypothetical value of 0 was used to determine statistical significance. The limit of detection was used for the CFU output value in the case that no mutant or WT CFU were recovered in the experimental output. A *P* value of less than 0.05 was considered to be indicative of a statistically significant result for all of the experiments, and the analysis was performed using Prism 8 (GraphPad Software, La Jolla, CA).

### Data availability.

All of the source data for this study are provided with the manuscript.
